# Analysis of gut microecological characteristics and differences between children with biliary atresia and non-biliary atresia in infantile cholestasis

**DOI:** 10.3389/fcimb.2024.1402329

**Published:** 2024-06-13

**Authors:** Yajun Liu, Yuan Zhang, Cheng Guo, Muxia Li, Ye Wang, Lin Zhang

**Affiliations:** ^1^ Department of Pediatrics, Hebei Medical University Third Hospital, Shijiazhuang, China; ^2^ Office of Academic Research, Beijing Children’s Hospital Affiliated to Capital Medical University, Beijing, China

**Keywords:** biliary atresia, infantile cholestasis, gut microbiome, dysbiosis, 16S rDNA

## Abstract

**Introduction:**

In infants with cholestasis, variations in the enterohepatic circulation of bile acids and the gut microbiota (GM) characteristics differ between those with biliary atresia (BA) and non-BA, prompting a differential analysis of their respective GM profiles.

**Methods:**

Using 16S rDNA gene sequencing to analyse the variance in GM composition among three groups: infants with BA (BA group, n=26), non-BA cholestasis (IC group, n=37), and healthy infants (control group, n=50). Additionally, correlation analysis was conducted between GM and liver function-related indicators.

**Results:**

Principal component analysis using Bray–Curtis distance measurement revealed a significant distinction between microbial samples in the IC group compared to the two other groups. IC-accumulated co-abundance groups exhibited positive correlations with aspartate aminotransferase, alanine aminotransferase, total bilirubin, direct bilirubin, and total bile acid serum levels. These correlations were notably reinforced upon the exclusion of microbial samples from children with BA.

**Conclusion:**

The varying “enterohepatic circulation” status of bile acids in children with BA and non-BA cholestasis contributes to distinct GM structures and functions. This divergence underscores the potential for targeted GM interventions tailored to the specific aetiologies of cholestasis.

## 1 Introduction

Infantile cholestasis, a liver disorder stemming from impaired bile production, secretion, or excretion, presents multifaceted aetiology and nonspecific clinical symptoms, often leading to diagnostic challenges and treatment delays. Recent investigations have shown that the gut microbiota (GM) is closely related to bile acid metabolism (Li et al., 2022b; Paik et al., 2022; Fan et al., 2023). Imbalances in GM composition frequently accompanied by abnormal bile acid metabolism during cholestasis (Wang et al., 2019; Song et al., 2021a; Yang et al., 2022). Wang et al. (2019) identified enrichment of intestinal *Clostridium*, *Gemella*, *Streptococcus*, *Veillonella*, and the *family Enterobacteriaceae* in children with cholestasis. They observed that changes in GM facilitated the conversion of primary bile acids to secondary bile acids; however, the precise mechanism remained incompletely elucidated. In our previous investigation of gut microecology in children with cholestasis, a significant reduction in GM diversity was observed, with an enrichment of *Bifidobacterium*, *Bacteroides*, *Streptococcus*, *Enterococcus*, and *Staphylococcus* in their intestines. Further GM functional analysis revealed an augmented gene abundance related to lipid metabolism and disease-related pathways in these children (Guo et al., 2018). Subsequently, our study on the dynamic changes of intestinal microecology in children with biliary atresia (BA) before and after Kasai surgery uncovered distinct GM variations in children with BA before surgery. Specifically, increased abundance of *Bacteroides*, *Rothia*, *Defluviitoga*, and *Collinsella* was observed in BA patients with Bifidobacterium deficiency, while *Actinobacteria* abundance increased in BA patients with Bifidobacterium predominance. Furthermore, a close correlation was identified between the baseline GM structure of children with BA and GM changes and liver function after surgical treatment (Li et al., 2022a). Other researchers in the field of BA have also observed a disrupted GM structure in patients with BA, with several major pathogens such as *Klebsiella*, *Streptococcus*, *Veillonella*, and *Enterococcus* dominating the progression of BA disease (Song et al., 2021a). Song et al. (2021a) found a significant positive correlation between *Enterococcus* and lithocholic acid derivatives, while Yang et al. (2022) observed an imbalance in the GM of patients with BA, coupled with alterations in faecal bile acid composition. Although various scholars have elucidated the gut microecological characteristics of cholestasis, including patients with BA (Wang et al., 2019; Song et al., 2021a; Li et al., 2020; Wang et al., 2020), there are significant differences in microbiota composition due to varying aetiologies, particularly in patients with BA who cannot secrete bile acids into the intestinal lumen, thus lacking interaction with GM. Hence, the primary disparity between BA and non-BA cholestasis in children lies in whether existence enterohepatic circulation of bile acids interacting with GM. However, there remains a notable absence of comparative and differential analysis of GM among children with BA, children with non-BA cholestasis, and healthy infants. Therefore, this study aimed to further analyse the influence of the presence or absence of bile acid enterohepatic circulation on GM, based on the previous study of gut microecology in children with cholestasis and BA conducted by our research group. The aim is to identify potential characteristic microbiota profiles associated with BA and non-BA cholestasis, thereby laying the groundwork for future clinical diagnostic and microecological interventions aimed at modulating GM.

## 2 Materials and methods

### 2.1 Ethics statement

This study was approved by the Ethics Committee of The Third Hospital of Hebei Medical University (the registration number: 2017–009-1) in accordance with the principles of the Declaration of Helsinki. Informed consent forms were provided to and signed by the legal guardians of all participants.

### 2.2 Attender enrollment

Children with cholestasis who were admitted to the Third Hospital of Hebei Medical University and the Second Hospital of Hebei Medical University, as well as infants who underwent outpatient health examinations, between December 2019 and September 2021, were included in the study. Inclusion criteria for all infants required full-term delivery, aged ≤180 days, absence of complementary food introduction, and no history of allergic diseases such as food allergies, eczema, allergic gastroenteritis, as well as no digestive system disorders such as diarrhoea or constipation. Additionally, participants should have no family history of genetic diseases. Diagnoses of BA and non-BA cholestasis followed the evaluation guidelines for infant cholestasis established by the North American Society of Pediatric Gastroenterology, Hepatology, and Nutrition (Moyer et al., 2004; Fawaz et al., 2017). BA diagnosis relied on intraoperative cholangiography and hilar bile duct exploration.

Exclusion criteria encompassed a history of antibiotic, microecological agent, or proton pump inhibitor usage from birth to the faecal sampling period. Additionally, participants were excluded if they presented with infectious diseases or other systemic conditions (such as congenital heart disease, tumours, etc.), if their mothers had diabetes, hypertension, or chronic liver disease during pregnancy, or if their mothers had a prolonged history of special medications or microecological agents used during pregnancy or lactation.

The included infants were further divided into three groups: control group (healthy infants, n=50), BA group (BA, n=26), and non-BA cholestasis group (IC, n=37). Fresh stool samples were obtained from healthy control group infants during outpatient physical examinations, while samples from infants with cholestasis were collected the morning after admission, disposable sterile stool collection cassettes (with faecal preservation solution) (Stratec, Germany) were used for collection, transported in liquefied nitrogen transfer tanks, and frozen in -80°C refrigerator. The blood samples were collected from the participants, and hepatic function was examined using the blood autoanalyzer (Beckman Coulter AU5800, Brea, CA, United States). The clinical indices for the assessment of hepatic function consist of serum alanine aminotransferase (ALT), aspartate aminotransferase(AST), total bilirubin (TBiL), direct bilirubin (DBiL), indirect bilirubin(IBiL), Total bile acids (TBA), total cholesterol (TC), total protein (TP),albumin (ALB).

### 2.3 DNA extraction, library construction, and sequencing

Bacterial DNA was extracted from stools using the E.Z.N.A.^®^ Soil DNA Kit (Omega BioTek, Norcross, GA, United States) according to the manufacturer’s protocols. The V3-V4 region of the 16S rRNA gene was amplified by primers 338F and 806R, using the PCR kit (TransGenAP221–02, Peking). The quality of the PCR product was determined (Qubit, Thermo Fisher Scientific, Singapore), and it was then prepared for library construction (TruSeq DNA PCR-Free kit, Illumina, San Diego,CA, United States). Then the eligible libraries were paired-end sequenced as 300 (nt) reads using the Illumina MiSeq platform (Illumina, San Diego, CA, United States). Detailed sequencing procedures can follow the methodology outlined in our previous research on intestinal microecology (Guo et al., 2018; Li et al., 2022a). Subsequently, the sequencing data of qualified samples were retrieved for downstream bioinformatics analysis.

### 2.4 Bioinformatics analysis

The raw reads were filtered using Trimmomatic with default parameters (Bolger et al., 2014). Subsequently, the paired reads deemed qualified were merged into tags with a minimum overlap of 50 bases. These tags were then clustered into Operational Taxonomic Units (OTUs) with 97% similarity using vSEARCH (Rognes et al., 2016). Taxonomy classification of the OTUs was conducted using the Ribosomal Database Project 16S rRNA databases (Cole et al., 2014). Beta diversity was assessed via Bray–Curtis dissimilarity, calculated using the “vegan” package in R (version 3.4.1). Permutational multivariate analysis of variance (PERMANOVA) was employed to evaluate differences in beta diversity. Co-abundance groups (CAGs) were analysed based on their co-abundance correlations (Wu et al., 2021).

### 2.5 Statistical analysis

For comparisons of clinical categorical data, either the chi-square test or Fisher’s exact test was employed, while the Wilcoxon rank-sum test was used for comparing continuous variables between two groups. For comparisons involving multiple groups, the nonparametric Kruskal–Wallis H test was employed, followed by the *post-hoc* Dunn’s test. The *p*-values from multiple hypothesis tests were adjusted using the Benjamini and Hochberg method (*FDR* < 0.05). Correlation analysis between the relative abundance of CAGs and clinical indicators was conducted using the Spearman coefficient, with a selection criterion of an absolute value of *r* ≥ 0.3 and adjusted *p* < 0.05. Principal component analysis (PCA) was employed to reduce data dimensions based on the vegan package (Oksanen, 2010). The importance of candidate biomarkers was determined by sorting based on the Gini index using the “randomForest” package (Liaw and Wiener, 2002).

## 3 Results

### 3.1 Infant basic information and clinical liver function index analysis

**Figure 1:**
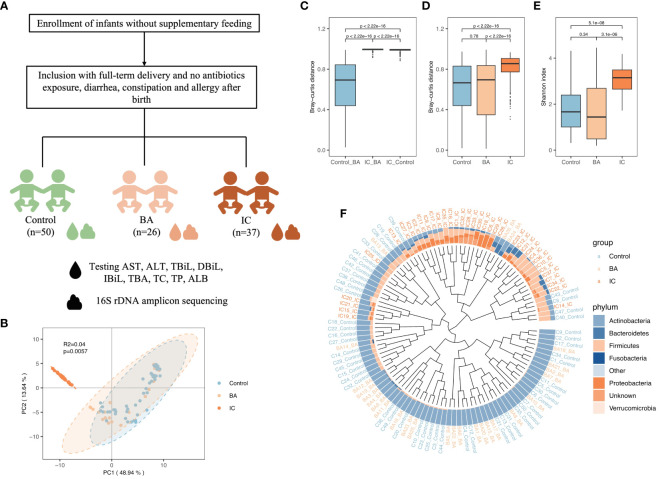
Overview of Gut Microbiota (GM) Structure in the Biliary Atresia (BA), Non-biliary Atresia (IC), and Healthy Control Groups. **(A)** Attender enrollment. **(B)** Principal component analysis plot illustrating the separation of microbial samples among the three groups. **(C, D, E)** Box plots highlighting inter-group beta-diversity, intra-group beta-diversity, and alpha diversity. **(F)** Dendrogram revealing the clustering based on the GM structure at the phylum level. Brown, orange, and blue names in the outermost part correspond to the IC, BA, and healthy samples, respectively.

A total of 113 infants were included in this study (**Figure 1A**), comprising 50 in the control group, 26 in the BA group, and 37 in the IC group. There were no obvious inter-group differences in age, gender, and delivery mode among the three infant groups (**Table 1**, **Supplementary Table S1**). Notably, the proportion of mixed feeding was significantly lower in the control group compared with the two other groups (**Table 1**).

**Table 1:** Sample information.

	Control(n=50)	BA(n=26)	IC(n=37)	*P*-value
**Age(d)**	54.34 ± 30.45	59.00 ± 20.31	52.14 ± 21.31	0.279
**Gender**				0.065
F	23	18	15	
M	27	8	22	
**Delivery mode**				0.651
CS	21	11	19	
ND	29	15	18	
**Feeding pattern**				<0.001
breast milk	26	12	27	
mixed	0	12	4	
formula	24	2	6	
Clinical indicator				
ALT(U/L)	14.2 ± 3.45	171.39 ± 146.79	265.94 ± 341.87	<0.001
AST(U/L)	23.66 ± 4.36	247.06 ± 162.66	289.71 ± 342.29	<0.001
TBiL(μmol/L)	8.57 ± 4.16	185.15 ± 59.37	136.64 ± 86.14	<0.001
DBiL(μmol/L)	3.1 ± 1.24	137.44 ± 38.07	93.74 ± 57.08	<0.001
IBiL(μmol/L)	5.49 ± 3.15	47.71 ± 27.64	42.89 ± 52.41	<0.001
TBA(μmol/L)	5.29 ± 2.39	174.62 ± 200.96	149.69 ± 232.60	<0.001
TC(mmol/L)	4.15 ± 0.50	5.59 ± 2.47	3.51 ± 0.99	<0.001
TP(g/L)	65.18 ± 4.33	58.34 ± 4.32	58.34 ± 4.32	<0.001
ALB(g/L)	48.11 ± 5.49	39.21 ± 3.20	37.81 ± 3.52	<0.001

BA, biliary atresia; IC, non-biliary atresia cholestasis; CS, caesarean section; ND, natural delivery; ALT, alanine aminotransferase: AST, aspartate aminotransferase; TBiL, total bilirubin; DBiL, direct bilirubin; IBiL, indirect bilirubin; TBA, total bile acids; TC, total cholesterol; TP, total protein; ALB, albumin.

Analysis of liver function indices revealed significantly elevated serum levels of ALT, AST, IBiL, and TBA in children from the BA and IC groups compared to those in the control group (*p* < 0.001). There was no statistically significant difference between the BA and IC groups (**Table 1**). Furthermore, serum TBiL and DBiL levels were markedly higher in children from the BA and IC groups compared to those in the control group (*p* < 0.001), with the levels in the BA group surpassing those in the IC group (*p* = 0.010, 0.003) (**Table 1**). Additionally, serum ALB and TP levels were significantly lower in children from the BA and IC groups compared to those in the control group (*p* < 0.001) (**Table 1**). Moreover, the serum TC level was higher in the BA group than in the IC group (*p* < 0.001) (**Table 1**). Further analysis showed that different feeding types had no effect on clinical liver function indicators (**Supplementary Table S2**).

### 3.2 Differences in GM structure between the control, BA, and IC groups

PCA, conducted using Bray–Curtis distance, revealed significant differentiation among microbial samples in the IC group compared to the two other groups (**Figure 1B**). Consistently, microbial samples from the BA and control groups exhibited minimal inter-group dissimilarity (*p* < 0.001, **Figure 1C**). Additionally, intra-group dissimilarity was notably higher in the IC group compared to the two other groups (*p* < 0.001, **Figure 1D**). Furthermore, infants in the IC group demonstrated the highest GM diversity (*p* < 0.001, **Figure 1E**). Regarding GM composition, Actinobacteria predominated in the GM of infants in the control and BA group (**Figure 1F**), whereas Firmicutes and Proteobacteria were prominent in the GM of infants in the IC group (**Figure 1F**).

### 3.3 Notable inter-group differences in CAGs

**Figure 2:**
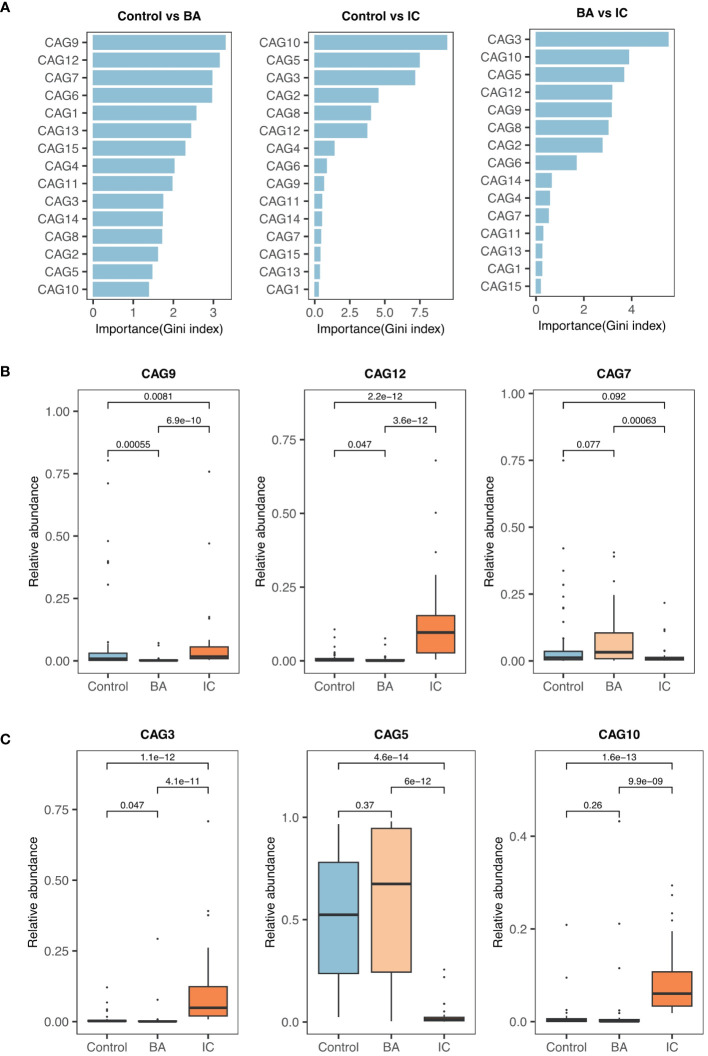
Selection and Comparison of Candidate Biomarkers among the Three Groups. **(A)** Ranking of co-abundance groups (CAGs) based on their contributions to the GM structure (Gini index). **(B)** Relative abundance of the top three significant CAGs (CAG9, CAG12, and CAG7) when comparing the biliary atresia (BA) and control groups. **(C)** Similarly, CAG3, CAG5, and CAG10 are the primary CAGs while comparing the non-biliary atresia (IC) and control groups and the BA and IC groups.

**Figure 3:**
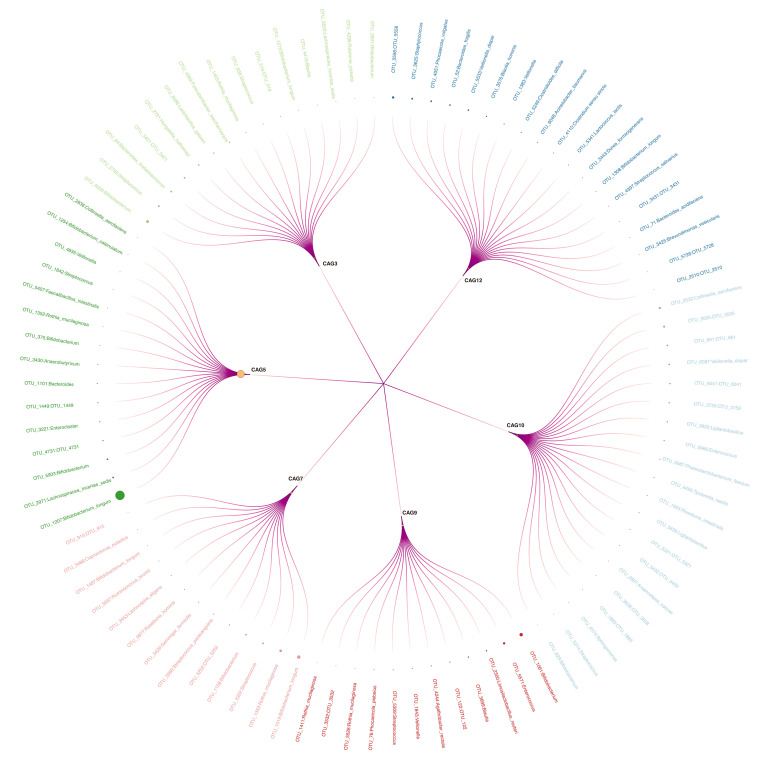
Circular Tree Map of Six Selected CAGs. Larger circle sizes indicate higher relative abundance. Outer labels with corresponding linkages represent Operational Taxonomic Units assigned to respective co-abundance groups.

Among 15 CAGs (**Supplementary Table S3**) identified, CAG9, CAG12, and CAG7 ranked as the top three discriminators between the BA and control groups (**Figure 2A**). Notably, CAG9 and CAG12 exhibited significantly lower abundance in the BA group (CAG9: BA 0.007 ± 0.018, IC 0.068 ± 0.143, control 0.081 ± 0.183; CAG12: BA 0.007 ± 0.018, IC 0.129 ± 0.145, control 0.009 ± 0.020; **Figure 2B**). In contrast, the relative abundance of CAG7 significantly decreased in the IC group (BA 0.085 ± 0.118, IC 0.021 ± 0.042, control 0.065 ± 0.136; **Figure 2B**). Further analysis revealed that CAG9 comprised 12 OTUs which were taxonomically annotated to *Bifidobacterium, Enterococcus, Limosilactobacillus reuteri, Blautia, Agathobacter rectalis, Veillonella, Streptococcus, Phocaeicola plebeius, Rothia mucilaginosa* and unknown taxa (**Figure 3**; **Supplementary Table S3**). CAG12 included 19 OTUs primarily classified into several genera, including *Bacteroides*, *Bifidobacterium*, and *Veillonella* (**Figure 3**; **Supplementary Table S3**). Conversely, CAG7, with a median abundance of 5.48%, comprised *Bifidobacterium longum*, *Rothia mucilaginosa*, and *Streptococcus* as its top three components (**Figure 3**; **Supplementary Table S3**).

CAG3, CAG5, and CAG10 emerged as the primary contributors to the inter-group differences in GM between the IC group and either the BA group or the control group (**Figure 2A**). Notably, CAG5 exhibited significantly lower abundance in the IC group (BA 0.572 ± 0.383, IC 0.030 ± 0.054, control 0.511 ± 0.312; **Figure 2C**). whereas levels of CAG3 and CAG10 were markedly higher in the IC group compared to the two other groups (CAG3: BA 0.016 ± 0.059, IC 0.104 ± 0.144, control 0.008 ± 0.020; CAG10: BA 0.032 ± 0.094, IC 0.089 ± 0.074, control 0.010 ± 0.312; **Figure 2C**). Among the 15 OTUs within CAG5, two lacked taxonomic annotation, while the remaining 13 were classified under *Bifidobacterium*, *Lachnospiracea*, *Enterocloster*, *Bacteroides*, *Anaerobutyricum*, *Rothia*, *Faecelibacillus*, *Streptococcus*, *Veillonella*, and *Collinsella* (**Figure 3**; **Supplementary Table S3**). *Bifidobacterium longum* notably dominated with a median abundance of 34.94% within CAG5 (**Figure 3**; **Supplementary Table S3**). CAG3 compromised 15 OTUs classified into various genera, including *Bifidobacterium* and *Bacteroides* (**Figure 3**; **Supplementary Table S3**). Similarly, CAG10 comprised 12 known components among 20 OTUs, classified into 12 different genera, such as *Collinsella* and *Veillonella* (**Figure 3**; **Supplementary Table S3**). Additionally, several other CAGs exhibited inter-group differences in relative abundance, including IC-accumulated CAG2, CAG4, CAG6, CAG8, CAG11, CAG14, and CAG15 (**Supplementary Figure S1**).

### 3.4 Association of CAGs with liver-related functional indicators

**Figure 4:**
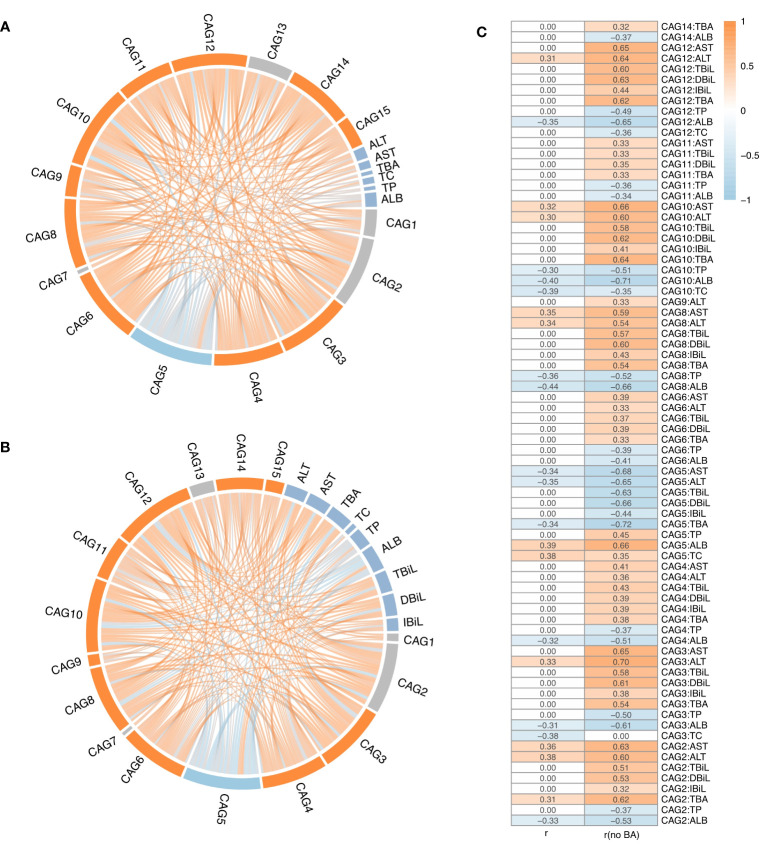
The correlation analysis between CAGs and liver function indices. **(A)** Correlations among 15 CAGs and liver function indicators across all the samples, or **(B)** after excluding biliary atresia (BA) samples. **(C)** Spearman’s coefficient (adjusted *p* value < 0.05) for all samples and BA-excluded samples. The ribbon represents the correlation between two elements, and the gradient of colours ranging from orange to sky blue represents correlation coefficients from 1 to -1.

Upon analysing all microbial samples, a significantly negative association was observed between IC-depleted CAG5 and 12 CAGs, including IC-accumulated CAG9, CAG12, CAG3, and CAG10 (*r* ≤ -0.3, adjusted *p* < 0.05, **Figure 4A**), but not with IC-depleted CAG7 (**Figure 4A**). Notably, IC-accumulated CAGs, which made substantial contributions to inter-group differences, exhibited notable positive associations with each other (*r* ≥ 0.3, adjusted *p* < 0.05, **Figure 4A**). Moreover, serum levels of AST and ALT correlated positively with IC-accumulated CAG2, CAG8, and CAG10, but negatively with IC-depleted CAG5 (|*r*| ≥ 0.3, adjusted *p* < 0.05, **Figure 4A**). Additionally, serum ALT levels were positive correlated with CAG3 and CAG12 (*r* ≥ 0.3, adjusted *p* < 0.05, **Figure 4A**). Further analysis revealed that IC-depleted CAG5 correlated negatively with serum TBA levels and positively with ALB and TC levels (|*r*| ≥ 0.3, adjusted *p* < 0.05, **Figure 4A**). Conversely, IC-accumulated CAGs correlated positively with serum TBA levels, but negatively with TC, TP, and ALB levels (|*r*| ≥ 0.3, adjusted *p* < 0.05, **Figure 4A**).

Subsequently, a re-analysis of the association between microbial samples in the control and IC groups was conducted, considering the lack of interaction between GM and bile acids whose secretion is impeded in BA. As hypothesised, more significant associations of CAGs with liver function indicators were observed after excluding microbial samples from the BA group (**Figures 4B, C**). For example, there were no significant associations between CAGs and serum levels of TBiL, DBiL, and IBiL when considering all microbial samples (**Figures 4A, C**). However, after excluding microbial samples from the BA group, serum TBiL, DBiL, and IBiL levels showed positive associations with IC-accumulated CAGs, including CAG2, CAG3, CAG4, CAG8, CAG10, and CAG12, as well as a negative association with IC-depleted CAG5 (|*r*| ≥ 0.3, adjusted *p* < 0.05, **Figures 4B, C**). Serum levels of TBiL and DBiL correlated positively with CAG6 and CAG11 (*r* ≥ 0.3, adjusted *p* < 0.05, **Figures 4B, C**). Furthermore, there were no significant associations between serum TBA levels and CAG3, CAG4, CAG6, CAG8, CAG10, CAG11, CAG12, and CAG14 when considering all microbial samples (**Figures 4A, C**). After excluding microbial samples from the BA group, serum TBA levels exhibited a notable positive correlation with the above CAGs (*r* ≥ 0.3, adjusted *p* < 0.05, **Figures 4B, C**), and the absolute value of Spearman coefficient associated with CAG2 and CAG5 doubled (**Figure 4C**). Similarly, the association between serum AST and ALT levels with CAGs became more evident after excluding microbial samples from the BA group (**Figure 4C**).

## 4 Discussion

Various factors can lead to cholestasis, including anomalies in biliary structure development, hereditary metabolic disorders, infections, and medication effects. Regardless of the underlying cause, liver damage induced by cholestasis shares common characteristics, such as compromised intestinal mucosal barrier function and disrupted “gut-liver axis” dynamics (Tilg et al., 2022). Disorders of liver and bile acid metabolism during cholestasis can affect the structure and function of GM via the “gut-liver axis” (Tilg et al., 2022). Additionally, GM contributes to maintaining the integrity of the intestinal mucosal barrier by producing antimicrobial peptides and short-chain fatty acids, thereby preventing intestinal bacterial translocation (De et al., 2022). When decompensation occurs, intestinal microecological imbalance emerges, accompanied by changes in bile acid metabolism. Given that children in the BA and IC groups exhibit distinct states of bile acid enterohepatic circulation, comprehending their respective GM characteristics and disparities between the two groups holds significant implications for future clinical biotargeted interventions targeting GM.

A growing body of research underscores the dysbiosis in GM among infants with cholestasis, hinting at the diagnostic potential of GM analysis (Guo et al., 2018; Li et al., 2020; Wang et al., 2020). Li et al. (2020) noted elevated levels of intestinal *Streptococcus* and *Lactobacillus* in children with cholestasis, which normalised after treatment, aligning with healthy infants. This suggests *Streptococcus gallolyticus*, *Parabacteroides distasonis*, and *Lactobacillus gasseri* as promising biomarkers for infantile cholestasis. Wang et al. (2020) identified the *Streptococcus*/*Bacteroides* abundance ratio as a biomarker distinguishing children with BA from healthy infants. Previous studies from our group revealed a more intricate GM co-occurrence network in children with cholestasis, with *Phyllobacterium*, *Ruminococcus*, and *Anaerostipes* as core nodes. *Rothia*, *Eggerthella*, *Phyllobacter*, and *Blautia* emerged as high-accuracy biomarkers distinguishing infants with IC from healthy infants. Moreover, a significant correlation between GM composition and liver function indicators was observed (Guo et al., 2018). Subsequently, our dynamic study on the intestinal flora of patients with BA delineated significant differences before and after Kasai surgery, the preoperative differences in GM affected postoperative liver function and GM recovery, indicating *Akkermansia* as a promising probiotic candidate (Li et al., 2022a). Building upon our previous research, this study delves into Guild analysis of GM in BA and IC children in cholestasis. CAG7, CAG9, and CAG12 emerged as pivotal in distinguishing patients with BA from healthy infants, while CAG3, CAG5, and CAG10 showed significance in distinguishing patients with IC from patients with BA or healthy infants, potentially serving as diagnostic biomarkers. Notably, microbial samples exhibited more pronounced abnormal GM in patients with IC compared to patients with BA. The divergence stems from the typical interplay between GM and bile acids in the intestine, influencing each other. GM modifies the bile acid spectrum, regulating its metabolism, thereby inhibits GM growth through direct or indirect effects and regulates its structural composition. Given the distinct bile acid enterohepatic metabolism states in children with IC and BA, whether existence an interaction between bile acids and GM underlies the fundamental reason for differential GM composition. Sun et al. (2022) similarly found disruptions in bile acid enterohepatic circulation precipitating GM diversity disorder, resulting in disparities in microbial community structure. Song et al. (2021b) observed that bile acid discharge into the intestine normalised after liver transplantation, curbing pathogenic bacterial growth by lowering intestinal pH, thereby affecting GM composition.

This study revealed that CAG3, CAG5, and CAG10 played a significant role in distinguishing between the IC group and the BA or control group based on GM inter-group differences, with CAG5 exhibiting notably lower abundance in the IC group. Within CAG5, among the 15 OTUs identified, two lacked taxonomic annotation, while the remaining 13 were classified as *Bifidobacterium*, *Lachnospiracea*, *Enterocloster*, *Bacteroides*, *Anaerobutyricum*, *Rothia*, *Faecelibacillus*, *Streptococcus*, *Veillonella*, and *Collinsella*. Of note, *Bifidobacterium longum* predominated in CAG5, aligning with findings from Zhou et al. (2019). *Bifidobacterium longum*, known as a beneficial bacterium in humans, modulates host immune responses, suppresses inflammation, and restores intestinal mucosal barrier function (Chichlowski et al., 2020; Yao et al., 2021; Mills et al., 2023). Recent research has also highlighted that metabolites of *Bifidobacterium longum*, such as 5-hydroxytryptamine, secondary bile acids, and short-chain fatty acids, mitigate liver inflammation, degree of fibrosis, and hepatocyte proliferation via the “gut-liver axis” (Yu et al., 2023). Moreover, *Bacteroidetes* can express enzymes involved in bile acid metabolism, such as bile salt hydrolase, which facilitates the breakdown of primary conjugated bile acids into free bile acids in the intestine, contributing to the metabolism of primary bile acids and the production of secondary bile acids in the intestine (Gérard, 2013; Wahlström et al., 2016). *Veillonella*, a bile acid-sensitive bacterium, demonstrates significant enrichment when bile acids decrease, and is associated with liver damage (Loomba et al., 2021). In conclusion, the contribution of CAGs varies across different diseases, depending on the OTU composition within CAGs and their functional status. It is believed that changes in CAGs might affect the disease status, liver function, and bile acid metabolism of children with BA and IC through diverse mechanisms.

This has been confirmed in numerous studies that GM is involved in the metabolism of bilirubin (Tabibian et al., 2016). Through correlation analysis of CAGs and corresponding liver functional indicators, this study observed that, based on microbial samples from all children with cholestasis, CAGs exhibited no correlation with bilirubin metabolism indicators such as TBiL, DBiL, and IBiL. However, after excluding the microbial samples from the BA group, a significant correlation between CAGs and bilirubin metabolism indicators emerged. This confirmed that in children with BA, where enterohepatic is completely disrupted, bilirubin cannot be excreted into the intestine with bile, thereby rendering GM involved in bilirubin metabolism ineffective. Additionally, it was confirmed that GM participates in bilirubin metabolism and influences damaged biliary tracts and hepatocytes via the gut-liver axis (Tajeddin et al., 2016). Furthermore, a positive correlation was observed between IC-accumulated CAGs and serum levels of AST, ALT, TBiL, DBiL, IBiL, and TBA, indicating that GM imbalance might contribute to liver injury occurrence, consistent with our previous findings (Guo et al., 2018). This underscores the involvement of GM-bile acids interaction in liver injury processes (Yang et al., 2022).

This observational study has certain limitations. First, the specific roles of the identified CAGs remain unclear, necessitating further investigation into how these CAGs influence bile acid metabolism. Second, due to the use of 16S rDNA sequencing technology, species analysis and functional interpretation of GM were not possible. Third, the potential suitability of the strain components within CAG5 as candidate probiotic strains for microecological interventions warrants verification in future studies.

In conclusion, the discrepancies in GM between children with BA and non-BA cholestasis primarily stem from the distinct bile acid enterohepatic circulation status in these two groups. The potential application of the strain components within CAG5, as identified in this study, as candidate probiotic strains for microecological interventions warrants further validation. Moreover, these findings form a basis for targeted GM interventions in children with cholestasis arising from diverse aetiologies.

## Data availability statement

The datasets presented in this study can be found in online repositories. The names of the repository/repositories and accession number(s) can be found in the article/**Supplementary Material**.

## Ethics statement

The studies involving humans were approved by the ethics committee of The Hebei Medical University Third Hospital. The studies were conducted in accordance with the local legislation and institutional requirements. Written informed consent for participation in this study was provided by the participants’ legal guardians/next of kin.

## Author contributions

YL: Data curation, Formal analysis, Investigation, Methodology, Resources, Software, Validation, Visualization, Writing – original draft, Writing – review & editing. LZ: Conceptualization, Funding acquisition, Methodology, Project administration, Supervision, Writing – review & editing. YZ: Data curation, Formal analysis, Validation, Visualization, Writing – original draft. CG: Data curation, Formal analysis, Resources, Validation, Writing – original draft. ML: Data curation, Formal analysis, Resources, Validation, Writing – original draft. YW: Data curation, Formal analysis, Resources, Validation, Writing – original draft.
