# Supplementary figures and images for: Analysis of gut microecological characteristics and differences between children with biliary atresia and non-biliary atresia in infantile cholestasis
Source: Front Cell Infect Microbiol. 2024 Jun 13;14:1402329. doi: 10.3389/fcimb.2024.1402329 (PMC11212454; doi:10.3389/fcimb.2024.1402329)

**CAG1**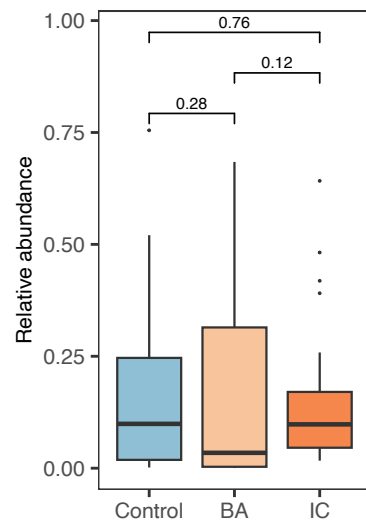**CAG2**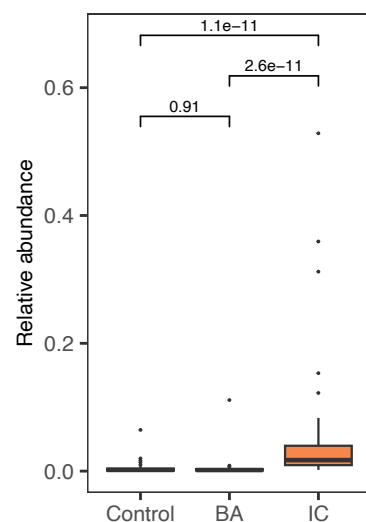**CAG4**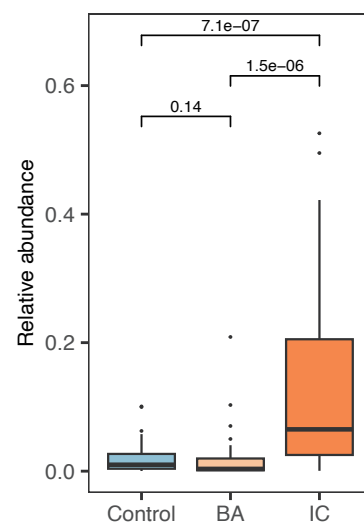**CAG6**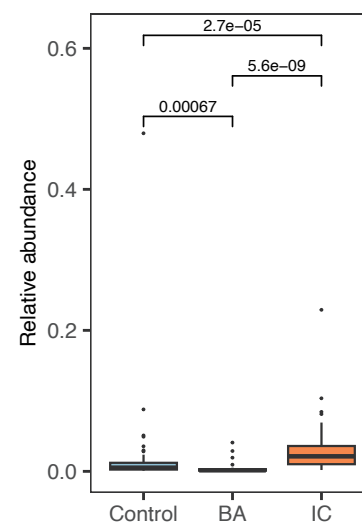**CAG8**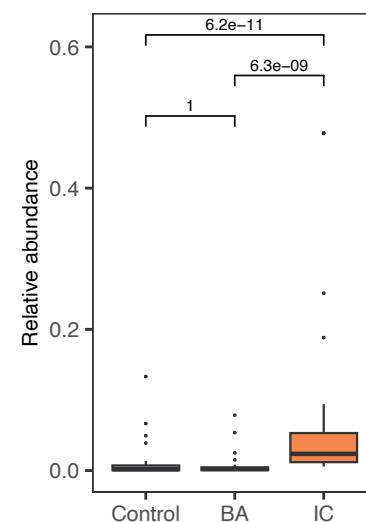**CAG11**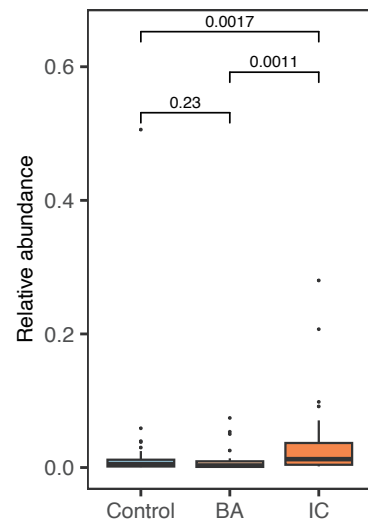**CAG13**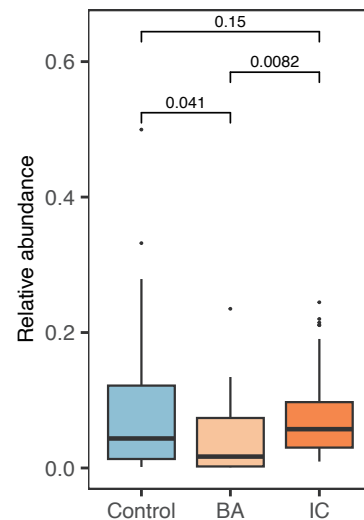**CAG14**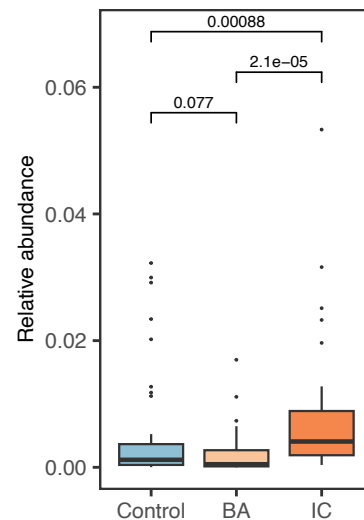**CAG15**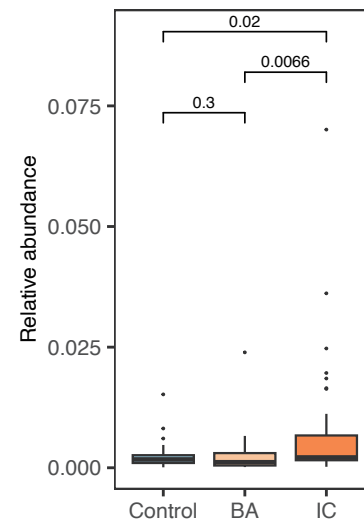

Supplement: Supplementary file 1 [file DataSheet_1.pdf]
